# Protein abundance profiling of the Escherichia coli cytosol

**DOI:** 10.1186/1471-2164-9-102

**Published:** 2008-02-27

**Authors:** Yasushi Ishihama, Thorsten Schmidt, Juri Rappsilber, Matthias Mann, F Ulrich Hartl, Michael J Kerner, Dmitrij Frishman

**Affiliations:** 1 Institute for Advanced Biosciences, Keio University, Tsuruoka, Yamagata 997-0017, Japan; 2 Center for Experimental BioInformatics, University of Southern Denmark, Campusvej 55, DK-5230 Odense M, Denmark; 3 Department of Genome-Oriented Bioinformatics, Wissenschaftszentrum Weihenstephan, Technische Universität München, D-85350 Freising, Germany; 4 Department of Proteomics and Signal Transduction, Max Planck Institute for Biochemistry, Am Klopferspitz 18, D-82152 Martinsried, Germany; 5 Department of Cellular Biochemistry, Max Planck Institute for Biochemistry, Am Klopferspitz 18, D-82152 Martinsried, Germany; 6 Center for Biological Sequence Analysis, BioCentrum, Technical University of Denmark, Kemitorvet 208, DK-1726 Lyngby, Denmark; 7 Wellcome Trust Centre for Cell Biology, University of Edinburgh, Edinburgh, EH9 3JR, UK; 8 Institute for Bioinformatics, GSF National Research Center for Environment and Health, Ingolstädter Landstraße 1, 85764 Neuherberg, Germany

## Abstract

**Background:**

Knowledge about the abundance of molecular components is an important prerequisite for building quantitative predictive models of cellular behavior. Proteins are central components of these models, since they carry out most of the fundamental processes in the cell. Thus far, protein concentrations have been difficult to measure on a large scale, but proteomic technologies have now advanced to a stage where this information becomes readily accessible.

**Results:**

Here, we describe an experimental scheme to maximize the coverage of proteins identified by mass spectrometry of a complex biological sample. Using a combination of LC-MS/MS approaches with protein and peptide fractionation steps we identified 1103 proteins from the cytosolic fraction of the *Escherichia coli *strain MC4100. A measure of abundance is presented for each of the identified proteins, based on the recently developed emPAI approach which takes into account the number of sequenced peptides per protein. The values of abundance are within a broad range and accurately reflect independently measured copy numbers per cell.

As expected, the most abundant proteins were those involved in protein synthesis, most notably ribosomal proteins. Proteins involved in energy metabolism as well as those with binding function were also found in high copy number while proteins annotated with the terms metabolism, transcription, transport, and cellular organization were rare. The barrel-sandwich fold was found to be the structural fold with the highest abundance. Highly abundant proteins are predicted to be less prone to aggregation based on their length, pI values, and occurrence patterns of hydrophobic stretches. We also find that abundant proteins tend to be predominantly essential. Additionally we observe a significant correlation between protein and mRNA abundance in *E. coli *cells.

**Conclusion:**

Abundance measurements for more than 1000 *E. coli *proteins presented in this work represent the most complete study of protein abundance in a bacterial cell so far. We show significant associations between the abundance of a protein and its properties and functions in the cell. In this way, we provide both data and novel insights into the role of protein concentration in this model organism.

## Background

Proteins fulfill a wide variety of functions and are central to almost all processes in living cells. In order to improve our understanding of the complex network of protein interactions in the cell, it is of central importance to obtain information about the activities of the individual components; these are directly linked to their cellular concentrations. The fast development of genomic and proteomic methods has already revealed the basic protein inventory of a few hundred different organisms, but large scale quantitative information on protein concentrations is still largely missing. Comprehensive analyses of cellular mRNA levels have proven to be highly useful tools to monitor the state of a cell, but by design they are missing all influences of the vast amount of posttranscriptional regulations. One of the few organisms where direct protein concentrations are available on a nearly proteome wide level is the yeast *Saccharomyces cerevisiae*. It has been subject to large scale protein quantification using epitope tagging of virtually the whole proteome followed by quantitative western blotting [1] and to single cell based quantitative proteomic analysis using flow-cell cytometry and a library of GFP-tagged yeast strains [2]. While both methods provided high-quality abundance data for nearly the entire proteome, their dependence on the availability of a strain library containing tagged versions of all proteins of interest presents a serious limitation. Depending on the organism under study, to generate such a library may involve an immense amount of work or may even be impossible to achieve.

The proteomics field and its key technology mass spectrometry are developing rapidly from qualitative towards quantitative measurements without the need for individual tagging of proteins. These efforts, however, are mostly restricted to the comparison of relative concentrations of the same proteins in different samples. Direct, non-relative abundance data of proteins, allowing a comparison of different proteins within and between samples, are still difficult to obtain on a large scale.

Mass spectrometry, in combination with protein and peptide separation methods, allows the efficient qualitative identification of proteins in complex mixtures. As an alternative to two-dimensional gel electrophoresis (2-DE) and mass spectrometric analysis of the resulting individual spots, shotgun approaches have been developed as suitable tools for large scale proteome analysis [3,4]. These are based on protease digestion of the sample as a whole and subsequent peptide separation and identification by multidimensional LC-MS/MS. However, in contrast to the 2-DE approaches, information about protein abundances is initially unavailable in the shotgun approaches. Relative quantification for abundance comparison of the same protein in different samples can be realized by incorporation of stable isotopes into the samples [5-7] which is utilized in methods like cICAT [8], iTRAQ™ [9], ^18^O-labeling [7] or SILAC [10]. Relative changes in concentration of the same protein between different experimental setups can be very accurately determined by these methods, but a major disadvantage is the absence of a direct measure of protein concentrations. Abundance comparison of different proteins is hence not possible.

Several mass spectrometric strategies have been reported to overcome this limitation. The more traditional ones utilize internal standards, e.g. spiking the complex mixture with peptides of known concentration [11,12], and typically require calibration for each protein to be quantified. A more recently introduced method describes a new parameter to express protein concentrations without the need of introducing labels or internal standards. It is calculated from the averaged ion intensities of the three most intense tryptic peptides per protein, as extracted from the ion current chromatograms. This parameter is called 'xPAI' for 'extracted ion intensity-based protein abundance index'. It has been shown to correlate well with known protein concentrations in the human RNA polymerase II complex [13] and rat mitochondria [14]. However, xPAI is limited to samples of low complexity since selection of only the three most intense peptides becomes unreliable with an increasing number of different proteins in the sample. Additionally, it is difficult to apply the xPAI approach to samples which were pre-fractionated at the peptide level, due to carry-over effects between the different fractions. A similar method has been described using an alternate scanning LCMS method (LCMS(E)), which is available on certain mass spectrometer instruments [15]. Here, all peaks in the MS spectra are selected as precursor ions for subsequent MS/MS scans resulting in lower peak intensity dependence of peptide identification as is the case for conventional data-dependent MS/MS scans. If the MS device allows this kind of detection mode it is preferable to xPAI, but it is still presented with the mentioned basic challenges of this approach.

Other label free ways of large scale protein quantification by MS make use of correlations between the number of actually identified tryptic peptides per protein and the theoretical number of tryptic peptides [16], or the molecular weight of the proteins [17]. These ratios have been termed 'protein abundance index' (PAI). More recently, we found empirically that PAI correlates better with the logarithm of protein concentration and defined an exponentially modified PAI (emPAI) [18]. Although such a method of concentration determination may not be expected to be overly precise, the accuracy of emPAI-derived concentration measurements has been shown to lie within an error range of only a factor of maximally 3.4 for 46 proteins in whole cell lysates of murine neuroblastoma (N2A) cells [18] and is therefore in the same range or better than protein concentration measurements based on staining methods. A major advantage is that the emPAI based protein concentration is automatically and quickly available for all proteins identified by MS without the need of any additional experimental setup. A similar approach was reported recently for the membrane proteome of *S. cerevisiae*, where protein concentrations were estimated by using the number of obtained spectra per protein divided by the length of the protein [19].

Determination of emPAI-based direct protein abundances can also be carried out in combination with some of the more accurate relative abundance measurement methods, e.g. iTRAQ, ^18^O-labeling or SILAC, since these do not introduce a detection bias towards certain peptides in the protease digested samples. ICAT, on the other hand, is dependent on the presence of a cysteine in a peptide in order for it to be detected, and cannot therefore easily be combined with the emPAI approach. The specificity to only a subset of all peptides renders this relative quantification method less well suited for concurrent direct protein quantification.

Protein identification in whole proteome analyses by mass spectrometry is still far from reaching complete coverage. Using state-of-the-art methods, up to ~50% of all expressed *S. cerevisiae *proteins could be identified by MS in a recent study [20], and it was concluded that current MS sensitivity and speed would still need to improve about tenfold to approach a proteome identification of 100%. Expected coverage should be a little higher for smaller proteomes and thus less complex samples. Nevertheless, to our knowledge, reported protein identification coverage values have not yet exceeded 61% for any proteome. The highest reported value so far was achieved by LC-MS analysis of the ionizing radiation-resistant bacterium *Deinococcus radiodurans *[21]. The study employed accurate mass and elution time tags to avoid time-consuming MS/MS events. The obtained coverage, however, was still far from complete, and, importantly, protein abundance information was absent. Here, we describe an approach to maximize MS based proteome identification coverage in an application to the *E. coli *cytosol, in combination with a reliable and quick concentration estimation of the identified proteins.

*E. coli *is a Gram-negative bacterium of the family Enterobacteriacae. Due to its simple cellular structure and the relative ease of its cultivation and biological modification, it has become the standard 'workhorse' of molecular biology, genetics and biotechnology. This resulted in *E. coli *becoming one of the most completely characterized organisms in biology. The genome of the laboratory strain *E. coli *K12 has been among the first organisms to be completely sequenced [22]. It has a relatively small size of ~4.6 Mb, and is predicted to code for approximately 4300 proteins. The genes, proteins, biochemical pathways and molecular interactions in *E. coli *have been subject to countless experimental studies and the growing number of available information in large scale databases like Genbank and Swissprot, but also in more specialized database projects like e.g. EcoCyc [23] or EchoBase [24] allows easy access to a wealth of information. However, in spite of the combined efforts of the scientific community, the complex network of molecular interactions within living organisms, including *E. coli*, is still far from being fully understood. Deciphering these interaction networks will be a major task of biology in coming years, and detailed knowledge about the concentrations of the individual parts in the system will be an important step on the way to accomplishing this goal.

Pioneering studies on two-dimensional electrophoresis of the *E. coli *proteome [25] were followed by 2-DE coupled MALDI-TOF approaches, which led to the identification of 381 *E. coli *proteins [26]. The first shotgun approach towards the identification of the *E. coli *proteome was reported by Gevaert *et al*. [27]. This study focused on methionine-containing peptides and identified approximately 800 proteins from an unfractionated *E. coli *lysate. It has, however, been suggested that such an approach may result in biased protein abundance data [13,17-19]. Corbin *et al*. [28] and Taoka *et al*. [29] then performed LC/LC-MS/MS approaches using multidimensional ion-exchange/reversed phase separation prior to MS/MS analysis. They reported protein expression profiling and protein abundance estimations, but based these purely on the number of identified peptides of each protein.

In order to extend the proteomic coverage of the *E. coli *cytosol and concurrently obtain minimally biased emPAI derived protein concentrations, we employed approximately 200 LC-MS/MS runs in combination with a variety of peptide/protein fractionation methods, different protease digestion schemes, LC-MS conditions and MS/MS fragmentation. Following this shotgun approach we identified more than thousand different proteins. We also report abundance data for these proteins based on emPAI, thereby providing the largest protein abundance set of the *E. coli *cytosol available to date.

## Results and Discussion

### Large scale determination of protein abundance in the *Escherichia coli *cytosol

We performed approximately 200 individual LC-MS/MS runs of the *E. coli *MC4100 cytosol, in combination with a variety of protein and peptide separation methods in order to maximize protein identification coverage. A summary of the methods employed in this study is given in Table 1 and described in detail in the Supplementary Information [see Additional file 1]. The decision to only investigate the cytosol of *E. coli*, rather than a whole cell lysate, was a direct consequence of our intention to provide reliable concentration estimates of all identified proteins, and avoid technical difficulties frequently arising from the quantitative proteolytic digestion of membrane proteins [28,30].

**Table 1:** Protein fractionation, peptide separation and mass spectrometric identification strategies for enhancement of proteome identification coverage explored in this study.

*Steps*	*Procedures*
(A) Protein fractionation	(1) SDS-PAGE slicing (2) Serial ultrafiltration (3) No protein fractionation
(B) Tryptic digestion	(1) In-solution digestion (2) In-gel digestion
(C) Peptide chromatography	(1) Strong cation exchange chromatograhpy (2) Strong anion exchange chromatograhpy (3) C18 ion pair chromatograhpy (4) PSDVB with NH_4_OH, using StageTip (5) No peptide chromatography
(D) Parent ion selection in LC-MS	(1) Simple repetition (2) Sequential static exclusion (3) Different ion pair reagents in subsequent runs (4) Subdivided scan range (5) Shallow gradient elution
(E) CID for MS/MS	(1) Quadrupole-TOF (2) Linear ion trap

Abbreviations: SDS-PAGE, sodium dodecylsulfate polyacrylamide gel electrophoresis; PSDVB, poly(styrene-divinylbenzene) copolymer; TOF: time-of-flight.

Evaluating the efficiency of the different protein and peptide separation methods and MS approaches listed in Table 1 (for details [see Additional file 1]), we found the following scheme to be optimal for our shotgun analysis of a cytosolic lysate of *E. coli *MC4100: Initial SDS-PAGE of the lysate sample with subsequent slicing of the gel lanes in five fractions was followed by in-gel tryptic digestion. The resulting peptide mixtures were subjected to strong cation exchange chromatography (SCX) (5 fractions, stepwise elution) and threefold ion pair chromatography (IPC) (60 min gradient) which was directly coupled to LC-MS/MS for peptide identification. Following this procedure with a quadrupole-TOF mass spectrometer, we identified a total number of 810 non-redundant proteins in a single *E. coli *cytosolic lysate sample. Including results of all previous runs during method comparison with this MS instrument type lead to the detection of a total of 1324 unique proteins of the *E. coli *cytosol. Note, however, that these numbers were preliminary and were based on a criterion where peptides with probability scores p < 0.05 and rank = 1 were temporarily accepted, even if only a single peptide was observed per protein. This acceptance criterion was subsequently strengthened to a minimum of two peptides per protein for compilation of the final list, as described in Materials and Methods.

We also performed experiments with a linear ion trap (LIT) with faster scan cycles. Parent ion selection with this device differed from the quadrupole-TOF instrument, leading to an increased applicable m/z range (Supplementary Table S1 [see Additional file 1]). Measurements with unfractionated samples of the *E. coli *cytosol revealed a considerably better performance of LIT when compared to quadrupole-TOF, as shown in Supplementary Figure S1 [see Additional file 1]. Combining the results from both types of MS instruments with protein and peptide pre-fractionation [see Additional file 1] further improved identification coverage and resulted in a total of 1655 proteins of the cytosolic lysate of *E. coli *MC4100, grown in rich medium.

**Figure 1:**
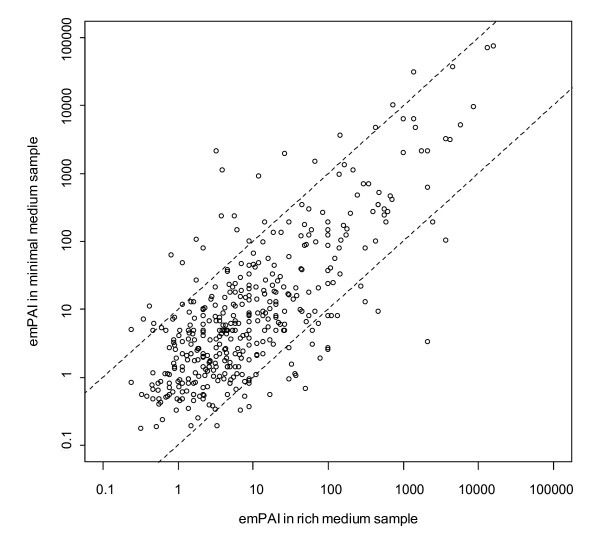
**Comparison of protein abundances in the *E. coli *cytosol of cells cultured in minimal and rich medium**. Protein abundance as derived by emPAI values. 454 proteins with more than two identified peptides were evaluated in samples from minimal and rich medium. The dashed lines indicate the positions equivalent to a concentration ratio of 0.1 and 10. The emPAI values in the minimal and rich medium correlate significantly with a Pearson correlation coefficient of 0.7 (pval < 10^-54^) and 0.77 (p-value < 10^-80^) for logrithmized variables.

**Figure 2:**
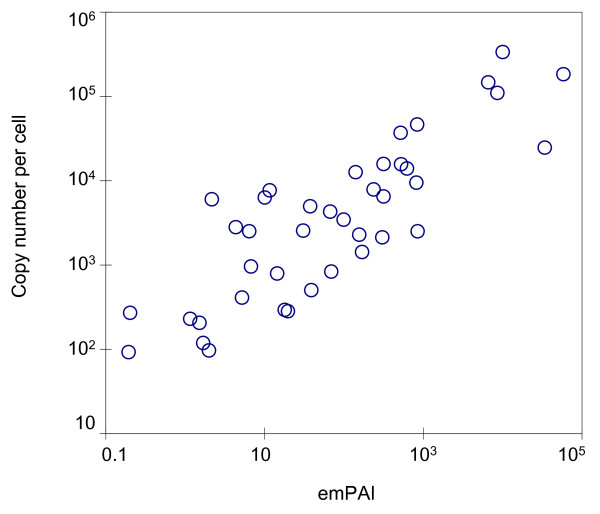
**Correlation between observed emPAI values and independently measured protein copy numbers per cell**. Protein abundances in the *E. coli *cytosol as measured by the emPAI approach correlate well with protein copy numbers per cell measured independently by isotope dilution using spiked *E. coli *BW25113 cells containing 40 proteins with known amounts [33]. A dynamic range of approximately 4 orders of magnitude of protein copy numbers per cell is covered. The Pearson correlation coefficient is 0.84 with a p-value < 10^-10 ^for logarithmized and 0.52 (p-value < 10^-4^) for non-logarithmized variables.

In order to cover a wider range of growth conditions in our measurements of the *E. coli *cytosolic proteome, we also applied an identical fractionation and identification scheme to cytosolic samples of MC4100 cultured in minimal medium. This analysis resulted in identification of 1305 unique proteins. Protein abundance profiling using the emPAI approach [18] showed a good correlation between protein concentrations in the samples from rich and minimal media, as shown in Figure 1. The majority of proteins exhibited concentration ratios within 0.5 and 2 when measured from cells cultured in minimal and rich medium. Other groups have previously reported on the abundance of certain *E. coli *proteins under various growth conditions, such as minimal versus rich media or from different growth phases [26,31,32]. The observed differences in protein abundance were within a factor of ten, with only few exceptions. Considering the described error range of emPAI derived protein concentration determination [18] and that of our particular system (as described in the next section, Figure 2 and Supplementary Figure S5 [see Additional file 1]), we decided to combine our datasets obtained from rich and minimal media for further analysis. We furthermore applied more stringent criteria for final protein identification, as described in Materials and Methods, to reduce potential false positive identification.

This combined and more stringent dataset yielded a total of 1103 proteins, quantified by emPAI, based on 13469 observed peptides with unique parent ions (10339 unique sequences) from 209 LC-MS/MS runs with less than 5% false positive rate (see Supplementary Tables S2 [see Additional file 2] and S3 [see Additional file 3], for all identified proteins and peptides, respectively). Our measurements thus provide ~32 – 41 % coverage of the approximately 2680 cytosolic proteins in *E. coli*, depending on the exact definition of the cytosolic dataset, as defined in Materials and Methods.

### Validation of the emPAI-based protein abundance dataset

To test for potential biases in the peptide identification process we compared a number of physico-chemical properties of the observed peptides with all predicted peptides from the corresponding proteins. These parameters are expected to influence the peptide behavior during many of the employed fractionation and separations steps as for instance chromatography. As listed in Supplementary Table S4 [see Additional file 1], the two sets did not exhibit a significant difference in peptide length, mass, pI or hydrophobicity. Peptide identification should therefore not be largely influenced by the separation and fractionation methods, which is a basic requirement for valid estimation of protein abundance by the emPAI approach [18].

Independent measurements of emPAI values from biological replicates revealed a good reproducibility with a Pearson correlation coefficient of 0.78 (Supplementary Figure S5 [see Additional file 1]). To further validate the protein abundance values based on emPAI and also test for potential biases introduced by the protein and peptide fractionation schemes, we compared the emPAI based concentrations of 40 proteins from our final set with independently determined concentrations. This was achieved by isotope dilution with a lysate of the E. coli K12 strain BW25113, for which accurate concentrations of these 40 proteins are known [33] (see Methods section for details). As shown in Figure 2, emPAI correlates well with the copy numbers per cell of these proteins over a range of approximately four orders of magnitude, with a Pearson correlation coefficient of 0.84 and a p-value < 10^-10^. The achieved accuracy of emPAI derived protein abundance in E. coli is therefore similar to the reported values [18] and the employed protein and peptide fractionation schemes did not introduce a detectable bias for the tested 40 proteins.

**Figure 3:**
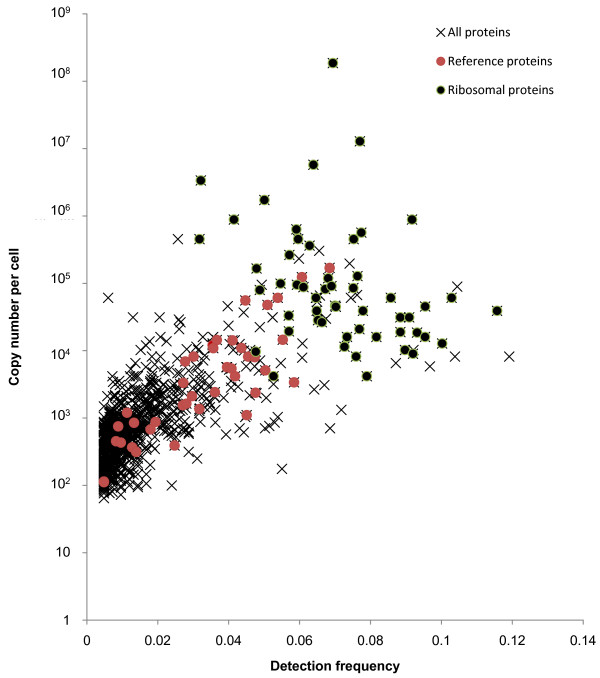
**Observed concentration and protein detection frequencies**. Correlation between the observed protein copy numbers (based on emPAI) and the detection frequency of the identified proteins. Detection frequency is defined as the average ratio of detection of the observed parent ions of a given protein in all performed LCMS experiments. Red dots indicate reference proteins (introduced in Figure 2), black dots indicate ribosomal proteins.

Proteins of very high abundance are expected to exhibit a saturated emPAI signal. In order to test the impact of this effect, we examined the correlation between measured protein concentrations and their detection frequency. This new measure was defined as the average detection ratio of the observed parent ions of a given protein in all of the 209 LCMS experiments. A high detection frequency indicates a possible saturation effect of the emPAI based concentrations of the affected protein. As shown in Figure 3, there is a good correlation between this measure and the emPAI derived protein concentration, yet with considerable noise in the high abundance and high detection frequency range. The measured concentrations of the reference proteins introduced in Figure 2 correlate well with their detection frequencies, while ribosomal proteins, which are some of the most abundant proteins in the cell, scatter noticeably. The saturation effect is responsible for the deviation of some ribosomal proteins to lower than expected observed concentration values. On the other hand, in particular the very short ribosomal proteins also deviate into regions with higher than expected measured concentrations. This can be explained by the small number of observable peptides of these proteins, which leads to higher errors of the emPAI signal, amplified by the high abundance of these proteins. Based on these observed high variations of the ribosomal protein concentrations we decided to remove all 53 detected ribosomal proteins from further analysis. There is a general tendency of other high abundance proteins and small proteins to exhibit emPAI concentrations of limited accuracy, but removal of all these proteins would inevitably lead to other artifacts in the following analysis. We therefore decided to keep these proteins and accept the noise they are introducing.

### Coverage of abundance measurements

In order to assess the coverage of our abundance measurements we compared the final set of 1050 proteins with a set of *E. coli *proteins known or predicted to be cytosolic. As shown in Table 2, the ratio of uniquely detected cytosolic proteins depends on the definition of this theoretical cytosol. As described in *Materials and Methods *we combined experimental localization data [34] with data from the PSORT database [35,36] and computational transmembrane segment predictions. Our cytosol definition – shown in the last row of Table 2 – results in 2680 theoretic cytosolic proteins that represent 62.5 % of the complete *E. coli *proteome. Applying the same strict criteria to the measured samples, 853 of the 1050 identified proteins (81.2%) can be safely considered cytosolic proteins. Under these very conservative assumptions we cover at least 32% (853 of 2680) of the theoretical *E. coli *cytosol. If, however, we extrapolate the experimental localization data we would cover ~75% (279 of 370) of the theoretical cytosol. Although the number of detected unique proteins that we do not consider as cytosolic is relatively high – 197 out of 1050, or 18.7% – their emPAI derived abundances indicate that these proteins represent only less than 5.4% of all measured protein copies in our sample. If the ribosomal proteins were not excluded, the amount of protein copies of non-cytosolic proteins would be less than 0.1%. This demonstrates the high specificity of our sample preparation and almost all proteins in the sample by mass can be considered cytosolic. Our method is highly sensitive in identifying and quantifying proteins even if they occur only in very low copy numbers. We were able to identify many proteins which are present in low copy number and are hardly detectable by other techniques. For example, the adenylyl protein glnE and members of the fts-family are known to be constitutively expressed at a very low level [37,38]. Overall, the abundance measurements for 1050 *E. coli *proteins presented in this work represent the most complete study of protein abundance in a bacterial cell so far, accounting for around one fourth of all *E. coli *gene products with a specificity of nearly 100% for the targeted cytosolic protein set.

**Table 2:** Comparison of the experimental cytosolic sample with the complete predicted *E. coli *proteome with respect to the number of predicted transmembrane segments (TMS), cellular localization from the PSORT-database and experimental localization data (EXP). Shown is the amount of unique proteins and the relation to the measured number of molecules in the cell.

*Attribute*^a^	*E. coli complete*		*Experimental cytosolic dataset*		
	
	Proteins ^b^	% Proteins^c^	Proteins^b^	% Proteins^c^	% Abundance ^d^
TMS = 0	3202	75.66	940	89.5	97.6
TMS = 1	265	6.26	50	4.8	1.7
TMS = 2	117	2.76	10	1.0	0.2
TMS = 3	54	1.28	7	0.7	0.1
TMS = 4	82	1.94	7	0.7	6.2E-02
TMS = 5	61	1.44	5	0.5	2.9E-02
TMS = 6	81	1.91	5	0.5	4.0E-02
TMS = 7	30	0.71	1	0.1	1.1E-02
TMS = 8	52	1.23	3	0.3	2.6E-02
PSORT = Cytoplasmic (C)	1574	36.51	554	52.8	65.3
PSORT = CytoplasmicMembrane (CM)	851	19.74	93	8.9	1.2
PSORT = Periplasmic (P)	142	3.29	61	5.8	1.6
PSORT = OuterMembrane (OM)	91	2.11	25	2.4	2.3
PSORT = Extracellular (E)	20	0.46	0	0.0	
PSORT = Unknown (U)	1577	36.58	288	27.4	29.0
PSORT = Unknown (multiple sites) (UM)	56	1.30	14	1.3	0.4
PSORT = C| CM | U | UM	4058	94.13	949	90.4	95.9
PSORT = C | U	3054	71.21	842	80.2	94.3
TMS = 0 & PSORT = C	1253	29.21	548	52.2	65.1
TMS = 0 & PSORT = C | CM	1903	44.37	580	55.3	65.7
TMS = 0 & PSORT = C | CM | U	3111	72.53	843	80.3	94.3
TMS < = 1 & PSORT = C	1335	31.13	553	52.7	65.3
TMS < = 1 & PSORT = C | CM	2033	47.40	592	56.4	65.8
TMS < = 1 & PSORT = C | CM | U	3334	77.73	877	83.5	94.8
TMS < = 1 & PSORT = C | U	2636	61.46	838	79.8	94.3
EXP = C	370	18.57	279	26.6	63.0
EXP = IM	76	3.82	46	4.4	4.7
EXP = OM	62	3.11	40	3.8	2.1
EXP = P	60	3.01	43	4.1	1.7
TMS < = 1 & EXP = C	281	6.55	279	26.6	63.0
TMS < = 1 & EXP = IM	62	1.45	42	4.0	4.6
TMS < = 1 & EXP = OM	44	1.03	36	3.4	2.0
TMS < = 1 & EXP = P	48	1.12	43	4.1	1.7
TMS < = 1 & (PSORT = C|U | EXP = C)	2655	61.90	853	81.2	94.6
(TMS < = 1 & PSORT = C|U) | EXP = C	2680	62.49	853	81.2	94.6

^**a **^Annotated attributes of the proteins depicted as logical statements. An ampersand (&) indicates that both conditions must be fulfilled ('*and*'), a vertical line (|) indicates '*or'*. The following abbreviations are used:

TMS – number of predicted transmembrane segments

PSORT – localization annotation from the PSORT database (C Cytoplasmic, CM Cytoplasmic Membrane, E Extracellular, OM Outer Membrane, P Periplasmic, U Unknown, UM Unknown – this protein may have multiple localization sites)

EXP – experimental localization data from [71] (C Cytoplasmic, IM Inner membrane, OM Outer Membrane, P Periplasmic)

^**b **^Number of unique proteins with the given attributes annotated

^**c **^Percentage of the unique proteins relative to the sum of unique proteins in the predicted *E. coli *proteome or in the experimental cytosolic sample, respectively

^**d **^Percentage of the actual number of protein copies found in the experimental sample, i.e. fraction of the total protein copy number sum.

### General characteristics of protein abundance in the *E. coli *cell

**Figure 4:**
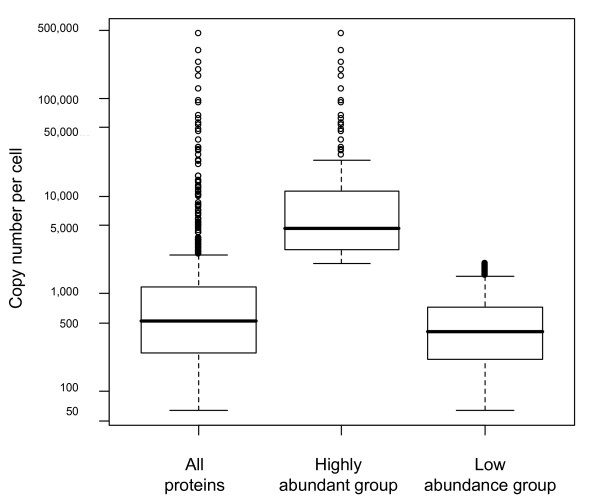
**Abundance distribution of all identified proteins**. Distributions are shown for the group of highly abundant proteins and the remaining low abundance protein group. Circles show distribution outliers as defined in *Methods*. The lower hinge represents the first quartile (25%) and the upper hinge the third quartile (75%). The high and low group were separated by clustering at a copy number cutoff of 2050 proteins per cell as described in *Methods*.

The main bulk of *E. coli *proteins in the cytosolic lysate are found in relatively small amounts, with 75% and 25% of them appearing in copy numbers below 250 and 1160, respectively (Figure 4). At the same time, a sizeable fraction of highly abundant proteins with copy numbers of up to 10^5 ^and more was identified. This broad dynamic range of abundance values corresponds to protein copy numbers per cell from ~100 to 10^5 ^and is in accordance with previously reported data on yeast in which the number of molecules per cell ranges from 50 to 10^6 ^[1]. Both *E. coli *and yeast proteins show an extreme value distribution, implying that this may be a general rule for abundance distribution in any cell. Due to the presence of very abundant proteins the arithmetic mean of the amount of copies per cell is around 3648 whereas the median copy number is only 526. The top 17% of abundant proteins are constituted by 179 proteins with more than 2050 copies per cell. The optimal separation between low and high abundance proteins at this threshold has been established by Expectation-Maximization clustering.

### Functional and structural classes

**Figure 5:**
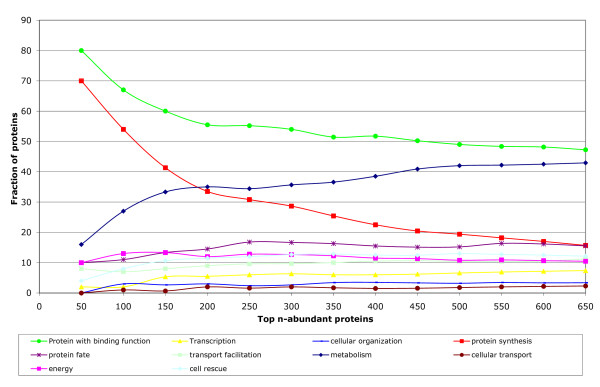
**Abundance functional profile**. Shown is the fraction of proteins which are involved in different functional categories in different abundance ranges. The first data point shows the functional breakdown of the 50 most abundant proteins, the second data point corresponds to the 100 most abundant proteins, and so on. Note that the fractions relative to the number of proteins (e.g. 50, 100...) do not sum up to 1 since a protein can have assigned multiple functions like *protein synthesis *and with *binding function*. The functional categories shown in the legend are the FunCat top level classifications as outlined in the Methods sections. In this plot all 1103 proteins – inclusive the 53 ribosomal proteins – are shown. Since the plot is based on relative ranking it is robust with respect to the observed copy number variability of these most abundant proteins.

In this section we compare whole groups of proteins with different functions and structures. Omitting the highly abundant ribosomal proteins would introduce a significant bias in these comparisons, with higher impact than the one caused by their less accurate emPAI based concentration values. For this reason all 1103 identified proteins, including the 53 ribosomal proteins, are considered. As expected, the latter are most abundant, followed by the proteins involved in metabolism (Table 3). In general, highly abundant proteins are predominately involved in *protein synthesis*, as shown in Figure 5. In the high abundance protein group (top 150 proteins) more than 40% of all proteins are involved in protein synthesis whereas in the low abundance group only 0.5% (42 of 915) are associated with protein synthesis processes. Other abundant functional groups are *energy *and *proteins with binding function*, while proteins associated with *transcription*, *transport *and *cellular organization *are relatively rare. In particular, transcription factors are found in small copy numbers since they act as regulatory elements and do not need to be expressed at high levels themselves, as discussed in [39]. In the low abundance group proteins involved in metabolism are predominant. In general, the distribution of functional roles among proteins of high and low abundance follows the pattern characteristic for predicted strongly and weakly expressed genes in bacteria [40]. With respect to enzymatic functions (Supplementary Figure S6 [see Additional file 1]), ligases, which play an essential role in protein synthesis, are the most abundant group, followed by isomerases. Oxidoreductases are the least abundant enzymes. Transferases and lyases are also not very abundant, but together they represent the majority of enzymes detected by our measurements. Structural fold occurrence among highly abundant proteins is also substantially biased. The most characteristic topology is represented by the *barrel-sandwich *fold (Table 4), as defined in the SCOP structural database [41]. The second most abundant fold is the *ribonuclease H-like motif *followed by the *OB-fold*. 55% (6 of 11) of proteins with the *ribonuclease H-like motif *belong to the *actin-like ATPase domain *superfamily associated with many metabolic reactions. Out of the 27 proteins with the *OB-fold*, 24 (or 87%) were assigned to the SCOP superfamily *nucleic acid-binding protein*, consistent with the finding that proteins involved in synthesis processes are the most abundant. This list of most abundant folds by protein concentration, as presented in Table 4, is in strong contrast to the fold distribution in bacteria, based solely on the number of different proteins in each group. Here, the five most common folds are the *Rossmann Fold, P-loop containing Hydrolase*, *Flavodoxin Like*, *TIM Barrel *and *Ferredoxinlike *fold [42]. With respect to protein concentrations in the cytosol, the *TIM-barrel, P-Loop containing Hydrolases*, and the *Ferredoxinlike *fold are found at places 7,8 and 11 of the list of most abundant folds. It is remarkable that proteins with the *P-loop containing Hydrolase *fold are on average about 10 times less abundant than proteins with the most abundant *Barrel-sandwich *fold. Furthermore, the widely spread *TIM-barrel *is on average around 6 times less abundant than the *Barrel-sandwich *fold. At the structural class level we found α/β proteins to be the least and α+β to be the most abundant. All-α proteins are the second most abundant proteins, followed by all-β proteins (data not shown). No significant correlation was found between abundance and the presence of structurally disordered regions.

**Table 3:** The most abundant functional groups in the *E. coli *cytosol.

***FunCat number***	***FunCat category description***	***Distinct proteins in this group***	***Rank (by mean copy number)***
05.01.01	ribosomal proteins	55	1
05.01	ribosome biogenesis	62	2
63.03.03	RNA binding	83	3
05	Protein synthesis	107	4
63.03	nucleic acid binding	144	5
40.03	cytoplasm	275	6
63	Protein with binding function or cofactor requirement (structural or catalytic)	483	7
63.07	structural protein	6	8
05.04	translation	34	9
63.01	protein binding	113	10
06.01	protein folding and stabilization	70	11
04.01.99	other rRNA-transcription activities	6	12

**Table 4:** The most abundant protein folds in the *E. coli *cytosol.

***Scop Fold***	***Number of distinct proteins with this fold ***^*a*^	***Rank (by mean copy number)***
Barrel-sandwich hybrid	10	1
Ribonuclease H-like motif	11	2
OB-fold	27	3
Thioredoxin fold	15	4
NAD(P)-binding Rossmann-fold domains	41	5
Transmembrane beta-barrels	12	6
Ferredoxin-like	22	7
TIM beta/alpha-barrel	47	8
Flavodoxin-like	28	9
DNA/RNA-binding 3-helical bundle	20	10
P-loop containing nucleoside triphosphate hydrolases	57	11
FAD/NAD(P)-binding domain	14	12
PLP-dependent transferases	14	13
Class II aaRS and biotin synthetases	13	14
Adenine nucleotide alpha hydrolase-like	17	15
Periplasmic binding protein-like II	22	16
ATP-grasp	10	17
S-adenosyl-L-methionine-dependent methyltransferases	12	18

^a ^All folds with 10 or more proteins were considered to avoid single outliers influencing the general trend.

### Protein aggregation

It has recently been shown that unfolded proteins with isoelectric points closer to neutrality and more stretches of alternating hydrophobic-hydrophilic residues with length 5 or more show increased aggregation rates *in vivo *[43,44]. Additional features associated with protein aggregation are protein length and hydrophobicity. Long proteins and more hydrophobic proteins are known to be more likely to aggregate [45]. Our analysis shows that highly abundant proteins have isoelectric points further away from neutrality and slightly fewer alternating hydrophobic-hydrophilic stretches in comparison to the low abundance proteins in *E. coli *as defined in the *Materials and Methods *section. Additionally we show that highly abundant proteins are on average shorter and less hydrophobic than proteins with low copy numbers (Table 5). Taken together, our data indicate that highly abundant proteins may have evolved to be less prone to aggregation. These observations are further strengthened when ribosomal proteins, known to be highly expressed, are also considered.

**Table 5:** Comparison of features associated with protein aggregation between high abundant proteins and the remaining detected proteins. The high abundant group is defined as described in Material and Methods.

*Property*	*Low abundant proteins Mean (Median)*	*High abundant proteins Mean (Median)*	*P-value KS-, MW-test*
Protein length (in amino acids)	386 (327)	309 (252)	10^-6^, 10^-7^
Number of alternating hydrophobic-/hydrophilic stretches (> = 5aa)	11.7 (9.0)	9.5 (8.0)	0.03, 10^-4^
pI distance from neutrality	1.52 (1.50)	1.69 (1,84)	0.003, 0.01
Hydrophobicity (Kyte-Doolite scale)	-0.20 (-0.21)	-0.25 (-0.24)	0.17, 0.08

### Amino acid composition

In agreement with Greenbaum *et al*. [39], greater frequencies of small amino acids Ala, Gly and Val were found in highly abundant proteins. Additionally we determined that Leu, Gln, Pro, Ser and Trp are more common in low abundance proteins whereas Lys and Glu is more common in the high abundance group. These compositional differences are a direct consequence of the functional bias observed in abundant and scarce proteins, as described above. Amino acid preferences in proteins of different functionality have been utilized before for coarse function prediction from sequence alone (e.g. [46]).

### Essentiality and length

**Figure 6:**
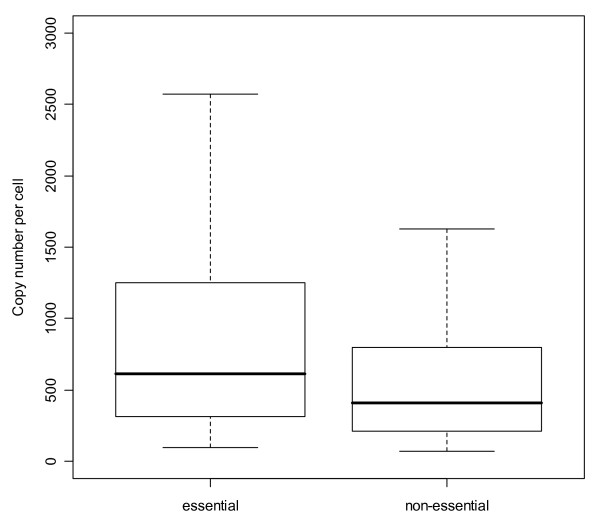
**Abundance and essentiality**. The abundance distribution of essential and non-essential proteins is shown: essential proteins are more abundant than non-essential proteins. The medians which represent 50% of all proteins within each group are shown as thick black bars, the one in the essential group is clearly higher (613 copies per cell *vs*. 432). Additionally in the essential group proteins can be found in higher abundance ranges than non-essential proteins (as can be seen by the difference of the upper whisker and upper hinge). A Mann-Whitney test as well as a Kolmogorov-Smirnov test indicated that the abundance distributions of essential and non-essential proteins are significantly different with p-values 0.0002 and 0.0001 respectively.

Protein abundance shows a remarkable correlation to the essentiality of a protein for bacterial growth, as determined by Gerdes *et al*. [47] (Figure 6). Low abundance gene products are overwhelmingly non-essential while highly abundant gene products tend to be predominantly essential. Furthermore, abundant proteins tend to be shorter (Supplementary Figure S7 [see Additional file 1]), similar to the trends reported for highly expressed genes in yeast [48,49].

### Protein abundance versus gene expression

The extent to which protein abundance correlates with the level of gene expression has been the subject of intensive studies in the past, primarily based on available yeast data. Early studies made on relatively small sets of abundance measurements were either inconclusive [50] or reported only a weak correlation between protein and mRNA abundance due to different rates of translation and protein degradation as well as various post-translational modifications [39]. In a more recent study Beyer *et al*. [51] hypothesized that a stronger correlation between mRNA and protein abundance may exist within functional modules such as "Metabolism", "Energy", and "Protein synthesis" and within cellular compartments.

**Figure 7:**
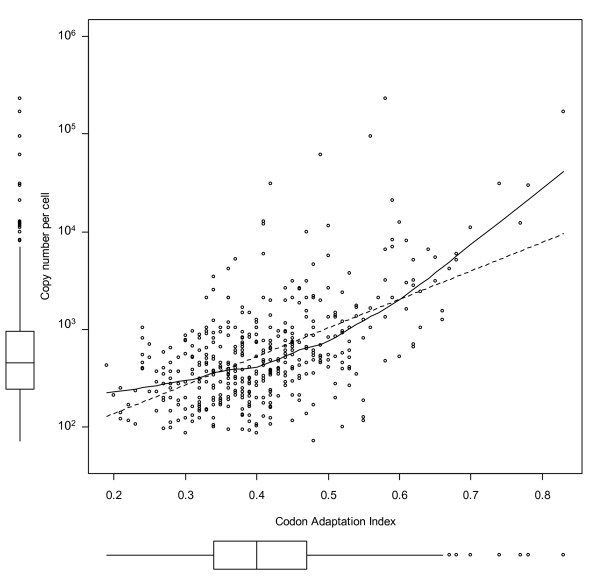
**Abundance versus codon adaptation index (CAI)**. Each point on the plot corresponds to a protein characterized by two values: abundance and CAI. The Spearman rank correlation coefficient r_s _between log-copy number and CAI is 0.5 and the Pearson correlation coefficient is 0.57 indicating a good non-random (p-values both < 10^-16^) correlation with some variance. The dotted line is a linear regression between log(copy number) and CAI, the solid line a loess local fitting curve.

**Figure 8:**
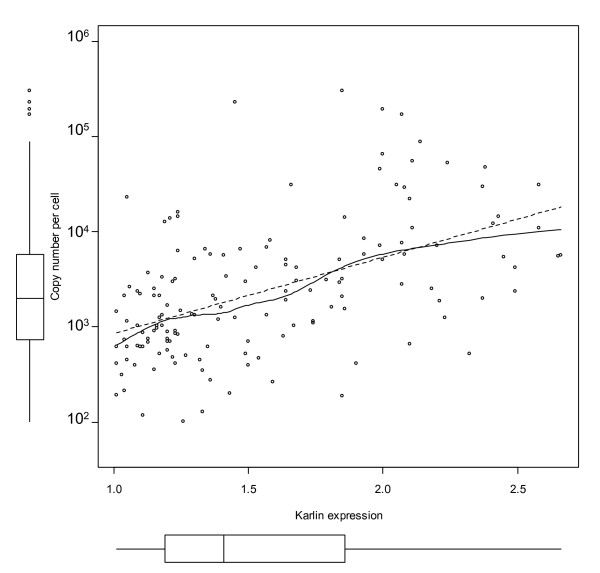
**Karlin's predicted gene expression and measured protein abundance**. The dotted line is linear regression and the solid line a loess local fitting curve. The Pearson correlation coefficient between log(copy number) and Karlin's expression value is 0.52 (p-value < 10^-12^) and the Spearman's rho is 0.53 (p-value < 10^-12^).

In this work we compare protein abundance with two computationally derived measures of gene expressivity. One of them, the codon adaptation index (CAI) as originally defined by [52] and refined by [53], has been shown to correlate both with mRNA expression levels and protein abundance in yeast [54]. The second expression measure is that of Karlin and co-workers [40] and is based on assessing codon usage difference between all genes and a subset of genes known to be highly expressed. Both CAI and the Karlin measure show a significant correlation with the emPAI values (Figures 7 and 8), although in the latter case the variance in the high abundance range was rather high.

**Figure 9:**
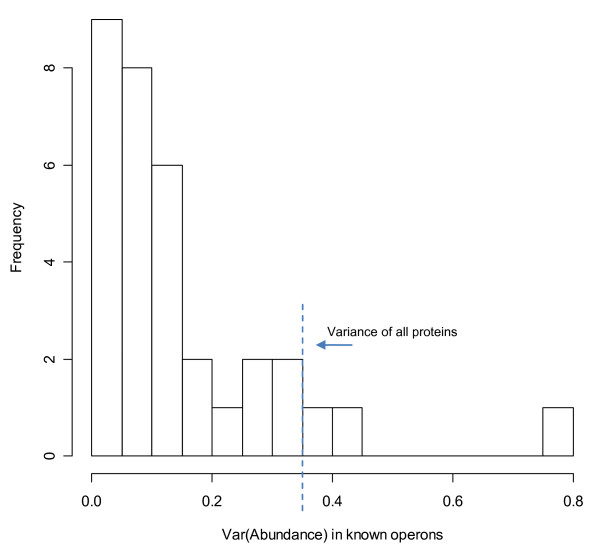
**Variance of abundance within known operons**. Only the 33 operons for which we have abundance data of 3 or more proteins are considered. The variance of all 1050 proteins is 0.35 and shown as dashed line. Low variance within an operon shows that the abundance of its proteins is similar. Here in 91% (30 of 33) of all operons the variance is lower than the variance of all proteins (left to the vertical bar). Copy number values are distributed according to the extreme value distribution and were therefore logarithmized for better representation.

Furthermore, the abundance variance within operons is smaller than the variance of all proteins in more than 90% of all known operons (Figure 9). Thus a large majority of proteins within the same operon display similar abundance values. This result is in accordance with what would be expected, since mRNA expression in prokaryotes mainly depends on the rate of transcriptional initiation. Assuming similar mRNA levels of genes within operons and comparable translation rates protein concentration mainly depends on the half-live of the proteins. The fact that in 9% of the operons the abundance variation is higher than expected shows the existence of additional mechanisms which control the level of protein expression.

## Conclusion

In this study we have developed a scheme to maximize the coverage of a proteomic study of the 'shotgun approach' in a reasonable timeframe and number of experimental steps. A combination of both protein and peptide separation methods before application to LC-MS/MS has proven to be the most efficient method to obtain a large and unbiased dataset. For the *E. coli *cytosol we found a combination of SDS-PAGE protein separation, strong cation exchange chromatography of the in-gel tryptic digest and LC-MS/MS with exchange of ion-pair reagents in subsequent runs to be most efficient. We show that our method is very sensitive to identify and quantify even proteins with extremely low copy numbers. For samples of different origin, the scheme would probably have to be slightly adapted, but it may serve as a good starting point for the experiments.

Calculation of the emPAI values from the mass spectrometrical data allowed us to obtain concentration information for all identified proteins and we therefore achieved to generate the most complete dataset on protein abundance in *E. coli *to date. Based on available experimental data as well as theoretical predictions of protein localization we estimate that our abundance measurements cover at least 32% of the *E. coli *cytosolic proteins by identity, with a contamination of non-cytosolic proteins of less than 0.1% by mass. The 197 identified proteins predicted not to reside in cytosol are all very low abundance proteins representing less than 5% of the protein copies of the cell even if the most stringent criteria are applied and ribosomal proteins are excluded.

Abundance of *E. coli *proteins strongly correlates with gene expressivity and displays a very broad dynamic range – from as high as 10^5 ^for molecular components of the biosynthetic machinery to a mere 65 typical for enzymes. There is also a marked bias in the occurrence of structural folds as a function of protein abundance. We found the *barrel-sandwhich*-fold as defined by the SCOP database to be the most characteristic topology for high-abundance proteins, while P-loop, TIM barrel, and Rossmann folds are associated with less copious gene products. Other essential traits distinctive for highly abundant proteins are less pronounced and include aggregation propensity and significantly higher chance to be essential.

## Methods

### Preparation of *E. coli *cytosolic lysate

*E. coli *MC4100 cells were grown at 37°C in rich or minimum medium to exponential phase (OD_600nm_~0.4), as described [55]. Lysis was induced by dilution of the spheroplasts into an equal volume of 25°C hypo-osmotic lysis buffer (50 mM Tris-HCl (pH 8), 0.01% (w/v) Tween 20, 10 mM MgCl_2_, 25 U/ml benzonase, 2 mM Pefabloc (Roche), 10 mM glucose and 20 U/ml hexokinase (Roche)). The supernatant was cleared at 30,000 × g for 10 min.

### Protein and peptide fractionation

See Supplementary Materials and Methods [see Additional file 1].

### NanoLC-MS/MS Analysis

See Supplementary Materials and Methods [see Additional file 1].

### Protein identification and abundance estimation

MS peak lists were created by scripts in Analyst QS (MDS-Sciex) or by Bioworks 3.1 (Thermoelectron) on the basis of the recorded fragmentation spectra and were submitted to the Mascot database searching engine (Matrix Sciences, London, UK) against the *E. coli *SwissProt database to identify proteins. The following search parameters were used in all Mascot searches: maximum of one missed trypsin cleavage, cysteine carbamidomethylation, methionine oxidation, peptide tolerance ± 0.2 Da for QSTAR data and ± 2.0 Da for LTQ data, MS/MS tolerance ± 0.2 Da for QSTAR data and ± 0.8 Da for LTQ data. All peptides with scores less than the identity threshold (p = 0.05) or a rank > 1 were automatically discarded. We also used the parent ion mass accuracy (mass deviation < 50 ppm for QSTAR data), the predicted retention times [56] (difference < 10 min), and protein molecular weight estimated from the gel slice as additional requirements for protein identification. Finally, using peptides within the above criteria, we only accepted proteins with two or more peptide hits. For decoy database searching, all peak lists were merged into two files to create QSTAR and LTQ peak lists. These merged peak lists were searched against a decoy database created by the Mascot script 'decoy.pl' supplied by Matrix Sciences. The obtained false positive proteins from two searches were merged and the final false positive rate was estimated to be 4.26% for the final protein identification list (containing a total of 1103 proteins).

Protein abundance expressed as emPAI scale was calculated using the number of observable peptides and the number of the observed parent ions. To calculate the number of observable peptides per protein, proteins were digested *in silico *and the obtained peptide masses were compared with the scan range of the mass spectrometer. In addition, the expected retention times under our nanoLC conditions were calculated according to the procedure of Meek [56] and Sakamoto *et al*. [57] with our own coefficients based on results of approximately 1500 peptides. Peptides that were too hydrophilic or hydrophobic were eliminated. In-house software was used to calculate emPAI values, the program is accessible at the Keio University web site. Redundancy of unique parent ions in the entire dataset was removed and the number of the unique parent ions per protein was counted. emPAI values were calculated as follows:

emPAI = 10^PAI ^- 1

$$\text{PAI}=\frac{N_{obsd}}{N_{obsbl}}$$

where N_obsd _and N_obsbl _are the number of observed parent ions per protein and the number of observable peptides per protein, respectively.

### Measurement of protein copy numbers per cell by isotope dilution

*E. coli *MC4100 cells were grown at 37°C in SILAC minimum medium containing Leu-D3 instead of Leu. A stock sample of unlabeled *E. coli *BW25113 cell pellet, including 59 enzymes with known amounts ranging from 9 to 70,000 copies per cell [33], was kindly provided by Drs. N. Sugiyama and K. Nakahigashi (Keio Univ). Based on total protein contents, these two samples were mixed at 1:1, 1:10 and 10:1, and were digested by trypsin. After desalting with C18-StageTip, each sample was analyzed with LC-MS/MS using QSTAR as described and was quantified by Mass Navigator version 1.2 (Mitsui Knowledge Industry, Tokyo, Japan). According to the dynamic range of the instrument, peptides with SILAC ratios of 0.1–10 were accepted for calculation of protein concentrations. A total of 40 proteins with at least two quantified peptides per protein were directly quantified from three samples.

### Genome data

Amino acid sequences of all proteins identified in this study were obtained from Swiss-Prot [58]. Throughout this work the primary Swiss-Prot accession code in conjunction with the Swiss-Prot entry name are used as unique protein identifiers. Codon Adaptation Index values (CAI) according to the method of [52] were used as reported by [22]. Classification of *E. coli *genes into three groups – (E) genes essential for cell growth (essential), (N) those dispensable for cell growth (non-essential), and (U) those unknown to be essential or non-essential – was based on the comprehensive experimental analysis of [47]. In the latter work, 630 genes were identified as being essential and 3126 as being dispensable using a genetic fingerprinting technique. Data on predicted expression measure of *E. coli *proteins [40] were downloaded from the Stanford University web server. Proteins possessing significant sequence similarity (BLAST [59] E-value threshold 0.001) to one or several domains of known three-dimensional structure as classified in the SCOP database [41] were attributed to the corresponding SCOP fold. Assignment of genes to functional roles as defined by the MIPS functional catalog version 1.3 [60] was conducted manually at Biomax Informatics AG. Where necessary, correspondence between published protein datasets and the SwissProt database was established based on sequence identity (at least 98%), with some ambiguous cases resolved manually. Minor discrepancies such as a missing methionine at the sequence start or a single amino acid replacement were tolerated.

### Coverage of the cytosolic protein content

To compare the coverage of our experimental cytosol sample with the theoretical protein content of cytosol we combined several recent sources of data as well as bioinformatics prediction techniques. For 13% (568 out of 4289) of *E. coli *proteins experimentally determined cellular localization information has been reported by Lopez-Campistrous *et al*. [34]. We further utilized the PSORT database [35] version 2.0 that provides localization annotation for 62% of the complete *E. coli *proteome (2678 proteins). The remaining *E. coli *proteins are classified in the PSORT database as "unknown" or "unknown with multiple possible localizations". We complemented this information with the number of transmembrane segments predicted using TMHMM [61] version 2.0.

Proteins with a high number of predicted transmembrane segments can be safely assumed to be not located within the cytosol. However the TMHMM predictions may lead to an over prediction of cytosolic proteins as this method reliably allows to exclude only those proteins that have multiple integral membrane segments. Furthermore, the possibility of falsely predicted membrane segments needs to be considered. We therefore combined the three data sources described above – the number of transmembrane segments, PSORT localization, and experimental localization – to find the most accurate definition of the *E. coli *cytosol proteome. First we consider all proteins that have at most one membrane predicted region and are annotated as "cytosolic" or "unknown" in the PSORT database. This criterion would predict 61.46% (2636 of 3289) of the *E. coli *proteome to be cytosolic (Table 2). The advantage of this estimate is twofold. On the one hand a false positive prediction of one membrane region is still tolerated and thus does not lead to loss of information. On the other hand the intersection with the independent PSORT data ensures that an over prediction of cytosolic proteins is avoided as much as possible. Finally we extend our previous definition and add all proteins that were experimentally determined as cytsolic proteins. This results in 2680 proteins that we adopt as our final estimate of the *E. coli *cytosol proteome. It is notable that the experimental localization data hardly increase the number of the defined cytosolic proteins (plus 1% or 44 of 2680 difference only). This shows the almost complete overlap of the first definition with the experimentally confirmed protein set and confirms the validly of our approach.

### Low vs. high abundance proteins

For convenience we considered proteins with copy number values greater than 2050 (emPAI > 29.0) highly abundant, while the rest of the proteins were attributed to the low abundance category. This optimal threshold was automatically found by clustering of the log-copy number values using the Expectation Maximization algorithm [62] as implemented in the WEKA machine learning workbench [63], version 3.5.6 using default parameters with the number of clusters set to two. As the copy number values are distributed according to the extreme value distribution, they were logarithmized to be useable with the Gaussian distribution approximation in the clustering process.

### Statistical methods

All statistical tests and most figures were prepared with the R software package version 2.0 and PROMPT [64]. To compare the distributions of two unpaired samples with non-Gaussian or unknown distributions, the rank-sum Mann-Whitney (MW) test and the two sample Kolmogorov-Smirnov (KS) test were applied using the significance threshold α = 0.05. The null hypothesis of the Mann-Whitney test is that the abundance means are equal. The null hypothesis of the Kolmogorov-Smirnov test is that the values of the two samples are drawn from the same continuous distributions. Both tests have the advantage that they make no assumptions about the distribution of data. To ensure that our tests are not biased by small sample sizes while comparing essential genes with their counterparts, the test results were verified with additional random sampling whereby each of the applied tests was repeated 10^5 ^times with a randomly drawn sample of the associated basic population. Then the p-value of the actual test was compared with the p-value distribution of random samples (data not shown). An observed p-value which lies in the 5% quartile shows a reliable test outcome independently of the sampling bias. Descriptive boxplot distribution statistics such as median, quartiles and outliers were generated with R. According to the canonical statistical definition, values greater than the 3rd quartile plus the inter quartile range (IQR) were considered outliers. The IQR is defined as the 3rd quartile value minus the first quartile value. Relationships between variables were analyzed utilizing the least squares regression, loess estimation and the Pearson or Spearman rank correlation methods implemented in R with default parameters.

### Operon structure

A set of known *E. coli *operons was obtained from RegulonDB [65]. For all operons with abundance information available for at least 3 proteins the variance of the natural logarithm of the emPAI values was calculated. The variance indicates how similar the abundance of the proteins within each operon is.

### Function and structure of proteins

Functional roles of gene products were described in terms of the manually curated hierarchical functional catalog (FUNCAT) [60]. In this catalog each of the 16 main classes (e.g., metabolism, energy) may contain up to six subclasses. An essential feature of FUNCAT is its multidimensionality, meaning that any protein can be assigned to multiple categories. Carefully verified manual assignment of *E. coli *gene products to functional categories was obtained from Biomax Informatics AG, Martinsried, Germany. Likewise, the SCOP database [41] provides a hierarchical classification of protein structural domains. SCOP fold assignments to gene *E. coli *products were based on BLAST E-value of 0.001. In this work both FUNCAT and SCOP designators were truncated to include only the two upper levels of hierarchy. Proteins assigned to the same SCOP fold were grouped and the average emPAI value for each group was calculated. To avoid individual outliers with very high or very low expression levels, only groups with 10 or more proteins were considered. The EC Enzyme Nomenclature information was taken from the Swiss-Prot protein descriptions.

Disorder predictions were taken from our PEDANT database where they are calculated with the software GlobPlot [66]. GlobProt utilizes the statistics of proteins known to have unstructured regions [67,68]. The number of alternating hydrophobic/hydrophilic stretches was computed as described [69]. The residues A, C, F, G, I, L, M, P, V, W and Y were considered to be hydrophobic and H, Q, N, S, T, K, R, D, E were considered hydrophilic in this study. The hydrophobicity of a protein was defined as $\frac{\sum_{i=1}^{n}H_{i}}{n}$, with *H*_i _denoting the hydrophobicity value of the amino acid at position *i *of a protein of *n *amino acids. Hydrophobicity values were calculated using the Kyte-Doolittle scale [70].

## Abbreviations

MS: mass spectrometry; (LC-)MS/MS: (liquid chromatography coupled to) tandem mass spectrometry; SDS-PAGE: sodium dodecylsulfate polyacrylamide gel electrophoresis; SCX: strong cation exchange; SAX: strong anion exchange; PSDVB: poly(styrene-divinylbenzene) copolymer; C18-DBAA: C18 reversed phase chromatography with di-n-butylamine acetate as a mobile phase additive; IPC: Ion pair chromatography; CID: collision induced dissociation; SILAC: stable isotope labeling by amino acids in cell culture; QTOF: quadrupole/time-of-flight; TFA: Trifluoroacetic acid

## Authors' contributions

YI performed all experimental procedures. YI, JR, FUH, and MM planned and designed the experimental part. YI, FUH, and MJK analyzed the MS data. TS and DF conducted all bioinformatics analyses. YI, TS, FUH, MJK, and DF wrote the manuscript.

## Supplementary Material

Additional file 1 Supplementary Information. Information from all the heterogeneous resources mentioned in the *Methods *section, supplementary results, supplementary Tables S1 and S4 and supplementary Figures S1 to S7. Click here for file

Additional file 2 Supplementary Table S2. Data on all identified proteins. Click here for file

Additional file 3 Supplementary Table S3. Data on all identified peptides. Click here for file
