# Supplementary material for: Protein abundance profiling of the Escherichia coli cytosol
Source: BMC Genomics. 2008 Feb 27;9:102. doi: 10.1186/1471-2164-9-102 (PMC2292177; doi:10.1186/1471-2164-9-102)
Supplement: Additional file 1 — Supplementary Information. Information from all the heterogeneous resources mentioned in the Methods section, supplementary results, supplementary Tables S1 and S4 and supplementary Figures S1 to S7. [file 1471-2164-9-102-S1.doc]

**Supplementary Information**

**Protein abundance profiling of the** **Escherichia coli cytosol**

Yasushi Ishihama, Thorsten Schmidt, Juri Rappsilber,

Matthias Mann, F. Ulrich Hartl, Michael J. Kerner, Dmitrij Frishman

**Contents:**

1. Supplementary Results.
2. Supplementary Tables S1 and S4.
3. Supplementary Figures S1 to S7.
4. Supplementary Materials and Methods.
5. References.

**1. Supplementary Results**

**Evaluation of different MS approaches and Protein and Peptide Fractionation Methods**

We first performed standard in-solution tryptic digestion of unfractionated *E. coli* cytosolic lysate, followed by 60 min LC-MS/MS analysis using a quadrupole-TOF instrument. This resulted in the detection of 944 unique peptides, representing 274 distinct proteins, in a single experiment. Consistent with previous findings [1], the number of newly identified proteins in eleven identical replications of this experiment quickly decreased (Supplementary Figure S2), resulting in identification of 484 proteins in total.

Exclusion of previously identified parent ions from analysis in subsequent runs (‘sequential static exclusion’, as opposed to ‘dynamic exclusion by data-dependent acquisition’), however, led to a marked increase of the number of newly identified peptides during subsequent LC-MS/MC runs [2] (Supplementary Figure S2). This method resulted in a total of 1840 identified peptides and 462 proteins in three successive runs, while simple reiteration without sequential static exclusion yielded only 1333 unique peptides and 359 proteins in three experiments. An even higher increase of peptide identification coverage (identification of 2097 unique peptides in three runs) was achieved with different ion-pair reagents (0.5% acetic acid, 0.02% trifluoroacetic acid and 0.02% heptafluorobutyric acid) in successive runs [3]. However, the 409 corresponding identified proteins did not exceed the results from sequential static exclusion. Presumably this is due to the fact that during sequential static exclusion the top four most abundant peaks of previous runs were excluded in order to select the less intense top five to eight peaks for MS/MS analysis and therefore the likelihood of identifying peptides of low abundance proteins increased. In runs with different ion-pair reagents, on the other hand, peptide retention times changed, but still the most abundant peak was selected for analysis, therefore favoring more abundant proteins. It is not possible to directly combine the two methods, since the exclusion list is partly based on the retention times in LC, which differ for the varying ion pair reagents.

Subdivided scan ranges for selection of precursors in only a certain mass range for fragmentation (*m*/*z* = 350–550, *m*/*z* = 550–750, or *m*/*z* = 750–1400, respectively)[4] did not result in improved peptide and protein identification (Supplementary Figures S2C and S2D). However, a standard experiment with a more shallow chromatography gradient (90 min) resulted in ~50% improvement of peptide identification (1525 peptides) and ~15% increase in identified proteins (354), but shallower gradients of more than 120 min performed poorly due to peak broadening, as had also been observed before [5]. Duplicate shallow gradient analysis with sequential exclusion or different ion-pair reagents did not provide superior data to triplicate analyses with the original gradient (data not shown) and was thus not explored further.

We chose two different ion exchange chromatography methods for peptide fractionation preceding C18 reversed phase based LC-MS/MS: strong cation exchange chromatography (SCX) with salt elution and strong anion exchange chromatography (SAX) with pH elution, as their separation principle is orthogonal to that of C18 reversed phase under acidic conditions. In addition to these ion exchange chromatography methods we employed two further approaches for initial peptide fractionation, C18 based chromatography with 0.1% dibutylammonium acetate (DBAA) as ion pair reagent, and reversed phase chromatography with a poly(styrene-divinylbenzene) copolymer (PSDVB) stationary phase under basic conditions (0.1% ammonium hydroxide). Three individual fractions were eluted in a stepwise fashion from disposable micro-columns of the StageTip format [6] in every experiment. Each fraction was analyzed by LC-MS/MS with a 60 min gradient in 0.5% acetic acid. As shown in Supplementary Figure S3, efficiency of peptide and protein identification improved considerably by initial peptide fractionation; the number of identified peptides was enhanced to ~140% and the number of identified unique proteins up to ~150% when compared to simple threefold LC-MS/MS repetition. All four StageTip fractionation methods resulted in similar improvement, PSDVB and SCX yielded just slightly more identified unique peptides and proteins than SAX and C18-DBAA.

We next tested for the effects of initial protein fractionation and different methods of tryptic digestion on peptide identification coverage. Initial protein separation is expected to be more effective than subsequent peptide fractionation for extension of the dynamic range of LC-MS/MS [5]. Here, we employed two approaches: sodium dodecyl sulfate-polyacrylamide gel electrophoresis (SDS-PAGE) and serial ultrafiltration. Following SDS-PAGE of the protein samples, complete lanes were cut out of the gel, sliced into fractions, and in-gel digested with trypsin. In ultrafiltration experiments, we iteratively used filters with decreasing cut-off sizes and directly subjected the obtained fractions to in-solution tryptic digestion. As expected, initial protein fractionation resulted in a considerably higher number of identified peptides and corresponding proteins than did sole peptide fractionation by SCX, the best performing peptide separation method (Supplementary Figure S3). Interestingly, the overlap of identified peptides with SDS-PAGE and SCX based separation constituted only ~200 peptides out of a total of ~1800, so a complementary use of SDS-PAGE and SCX appeared as the most sensitive way of protein and peptide fractionation before LC-MS/MS. A very similar picture emerged when the cytosolic sample was pre-fractionated by ultrafiltration and subsequently trypsin digested in solution (Supplementary Figure S3). Initial protein fractionation by ultrafiltration led to a significant enhancement of the peptide and protein identification coverage, and allowed detection of many peptides and proteins that would have stayed unidentified by just tailoring the method to enhance the separation of peptides, as shown in Supplementary Figure S3 for the example of online SCX peptide separation (see Supplementary *Materials and Methods* for details). The combinations of SDS-PAGE with in-gel tryptic digestion and ultrafiltration with in-solution tryptic digestion performed very similarly. This can presumably be attributed to a combinatorial effect of the lower separation power of ultrafiltration compared to SDS-PAGE, protein loss by irreversible binding of proteins to the ultrafiltration membrane, and more efficient tryptic digestion in solution than in gel [7].

Based on these results we found the following scheme to be optimal for analysis of the cytosolic lysate of *E. coli* MC4100: SDS-PAGE with subsequent slicing (5 pieces) / in-gel tryptic digestion / SCX (5 fractions, stepwise elution) / 3 x IPC (60 min gradient) coupled to LC-MS/MS.

**2. Supplementary Tables**

**Supplementary Table S1.** *Comparison of QSTAR and LTQ mass spectrometry.*

Conditions are as in Figure 4. Averaged values were calculated from 1532 and 1832 unique peptides measured by QSTAR and LTQ, respectively.

|  | *average peptide mass* | *average m/z* | *average charge* | *average retention time(min)* |
| --- | --- | --- | --- | --- |
| QSTAR | 1180 | 561 | 2.1 | 72.15 |
| LTQ | 1639 | 802 | 2.1 | 88.26 |

**Supplementary Table S4.** *Comparison of predicted peptides and observed peptides.*

| *Dataset* |  | *Length* | *Mass* | *pI* | *Hydrophobicity^1^* |
| --- | --- | --- | --- | --- | --- |
| observed | Mean | 13.28 | 1468.13 | 6.35 | -0.19 |
|  | Std. Deviation | 5.22 | 550.91 | 2.31 | 0.72 |
|  | Variance | 27.25 | 303501.83 | 5.34 | 0.52 |
|  | Minimum | 4.0 | 374.46 | 3.01 | -3.72 |
|  | Maximum | 47.0 | 5368.79 | 12.52 | 2.94 |
|  | Median | 12.0 | 1358.60 | 6.31 | -0.16 |
| predicted | Mean | 13.36 | 1493.17 | 6.19 | -0.15 |
|  | Std. Deviation | 5.05 | 531.98 | 2.19 | 0.79 |
|  | Variance | 25.54 | 283006.37 | 4.81 | 0.63 |
|  | Minimum | 5.00 | 799.00 | 3.01 | -3.67 |
|  | Maximum | 29.00 | 2799.30 | 12.98 | 2.99 |
|  | Median | 12.00 | 1371.48 | 6.22 | -0.12 |

^1^ Grand average hydrophobicity using the Kyte-Doolittle scale as described in *Material and Methods*.

**3. Supplementary Figures**

**
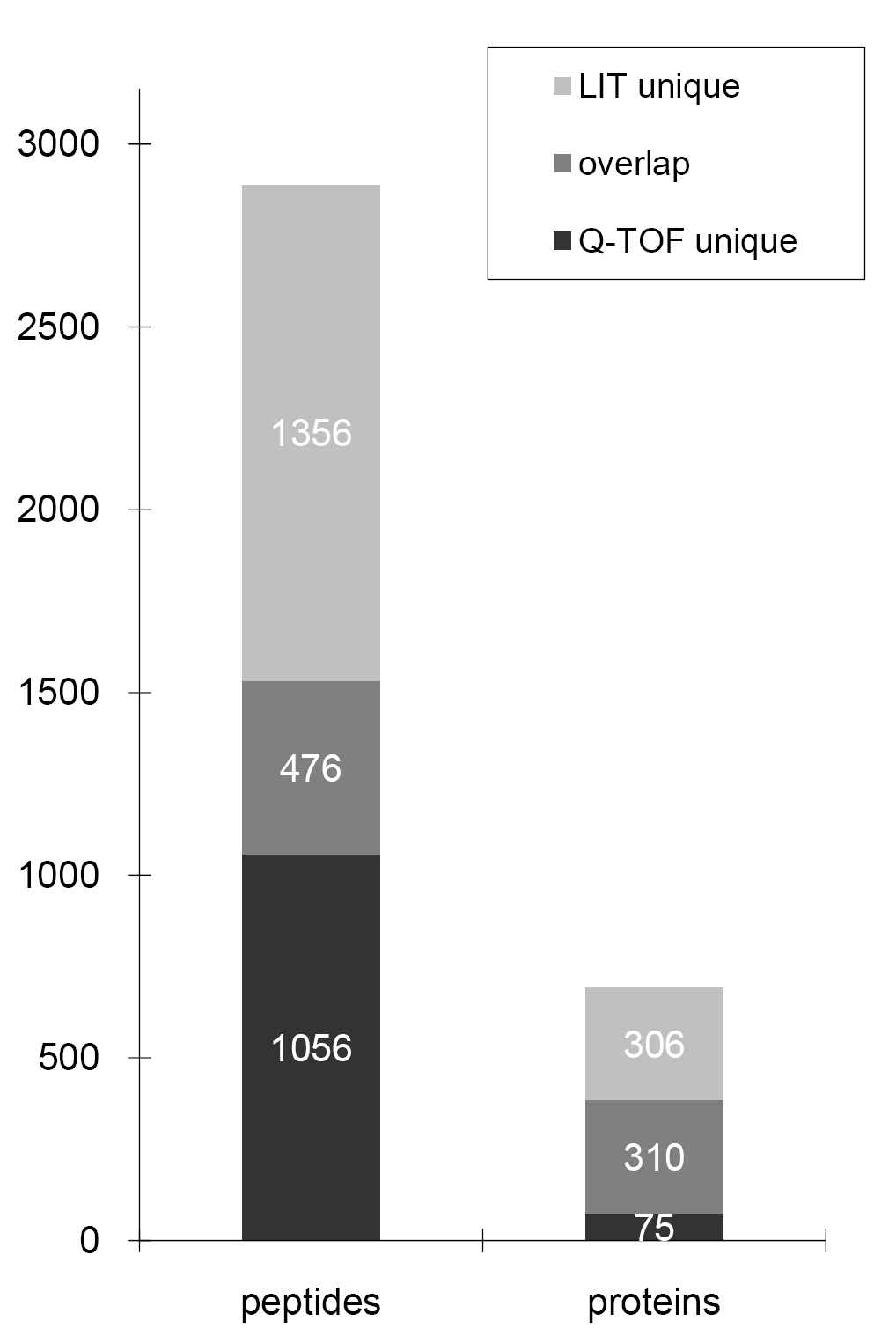
**

**Supplementary Figure S1.** *Quadrupole-TOF and linear ion trap (LIT) instruments in identification of peptides and proteins.*
LC-MS/MS was performed with a 90-min gradient and acetic acid as ion pair reagent. *E. coli* cytosolic samples were digested in solution and applied to LC-MS without further fractionation.

**
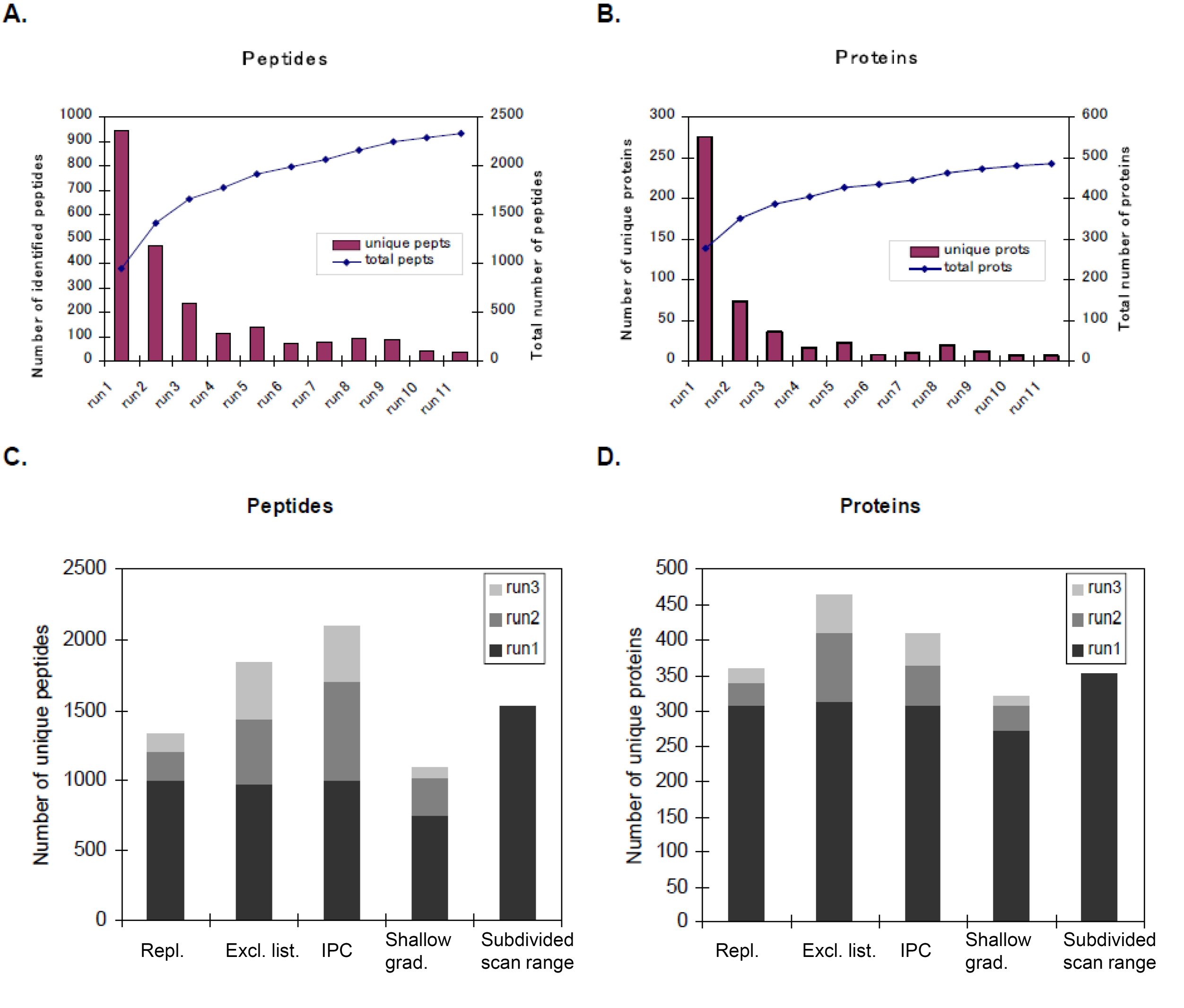
**

**Supplementary Figure S2.** *Replication of LC-MS/MS experiments with differing approaches for maximization of the number of identified proteins in unfractionated E. coli cytosol.***A and B:** Simple replication of identical LC-MS/MS experiments lead to a quick saturation of the total number of identified unique peptides **(A)** and, in particular, the correlating proteins **(B)**. 60-min gradient LC coupled to QSTAR mass spectrometry was performed with 0.5% acetic acid as ion pair reagent. 1.5 µg cytosolic E. coli proteins were digested in solution without any previous protein fractionation. **C and D:** As a control, a simple threefold replication with LC-MS/MS conditions as described for A and B is reported (Repl.). Sequential static exclusion (Excl. list) of parent ions in subsequent experiments using the same conditions resulted in a considerable increase of both identified unique peptides **(C)** and the corresponding proteins **(D)**. Exchange of the ion-pair reagents in subsequent runs (acetic acid, TFA and HFBA) resulted in an even higher amount of total identified peptides (IPC) **(C)**. This did not, however, result in the same increase in the identified unique proteins **(D)**.

**Supplementary Figure S3.** *Influence of initial protein and peptide fractionation on the performance of LC-MS/MS*.

Number of identified peptides **(A)** and proteins **(B) **following initial peptide fractionation. Stage Tip peptide fractionation with stepwise elution in three fractions was performed with the indicated chromatographic method before application to C18 reversed phase chromatography coupled MS/MS. The fractionated samples were measured on a QSTAR instrument coupled to 60-min gradient LC with acetic acid as ion pair reagent. *Abbreviations*: Repeat, simple threefold replication of the standard LC-MS/MS experiment as described for Figure 1 without initial peptide fractionation; SCX, strong cation exchange chromatography; PSDVB, poly (styrene-divinylbenzene) copolymer reversed phase chromatography; SAX, strong anion exchange chromatography; C18-DBAA, C18 reversed phase chromatography with dibutylammonium acetate as ion pair reagent.


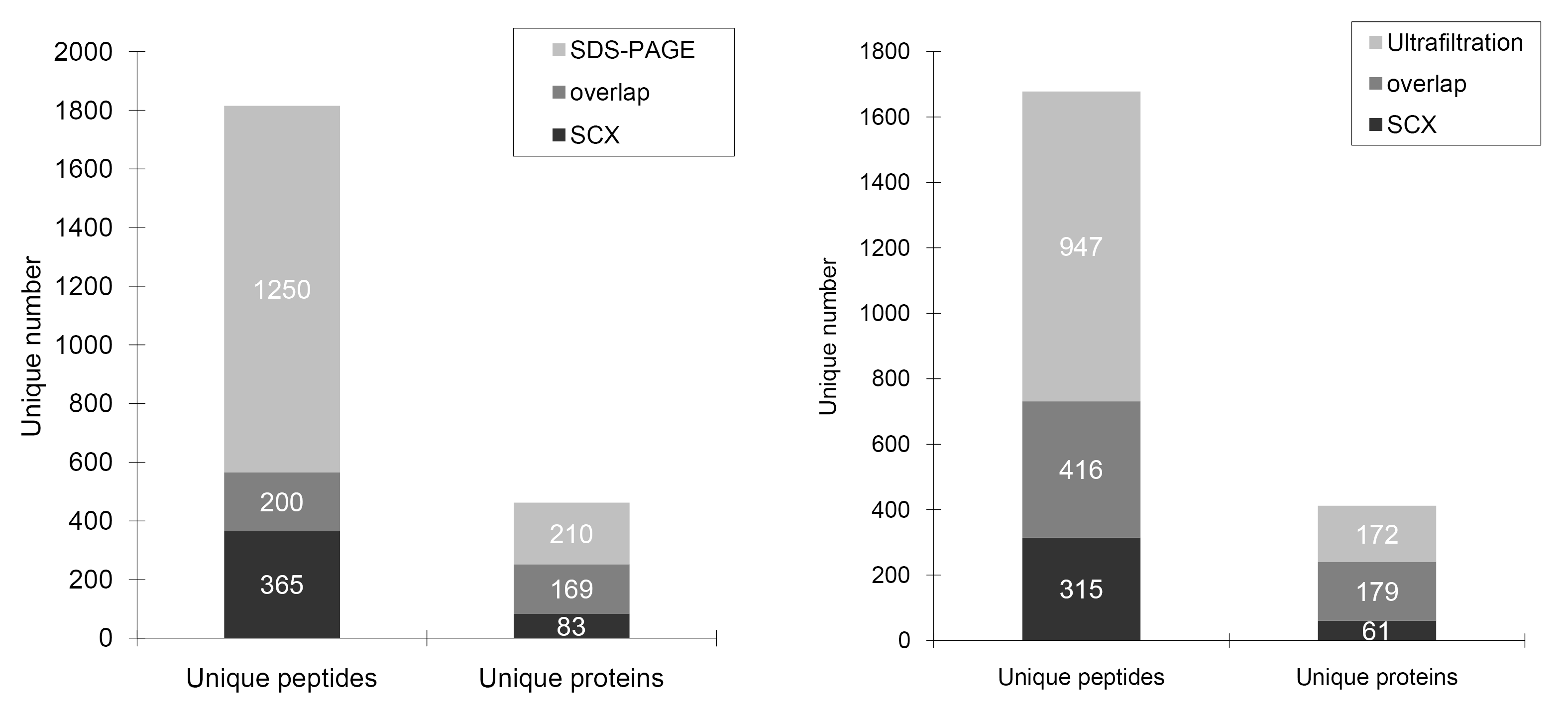


**Supplementary Figure S4.** *Comparison of initial protein separation to peptide separation methods on the performance of LC-MS/MS*.

Comparison of the number of identified peptides and proteins by MS upon on-line SCX peptide separation versus initial SDS-PAGE protein fractionation (5 fractions) **(A)** and upon StageTip based SCX versus Millipore ultrafiltration (4 fractions) **(B)**. LC-MS/MS was performed on a QSTAR instrument. LC with a 15 min gradient and acetic acid as ion pair reagent was used. *Note that due to a shorter gradient in LC the absolute numbers of identified peptides and proteins are not directly comparable between Figures 2 and 3. Furthermore, due to different fraction sizes, absolute numbers are not directly comparable between (A) and (B).*

**
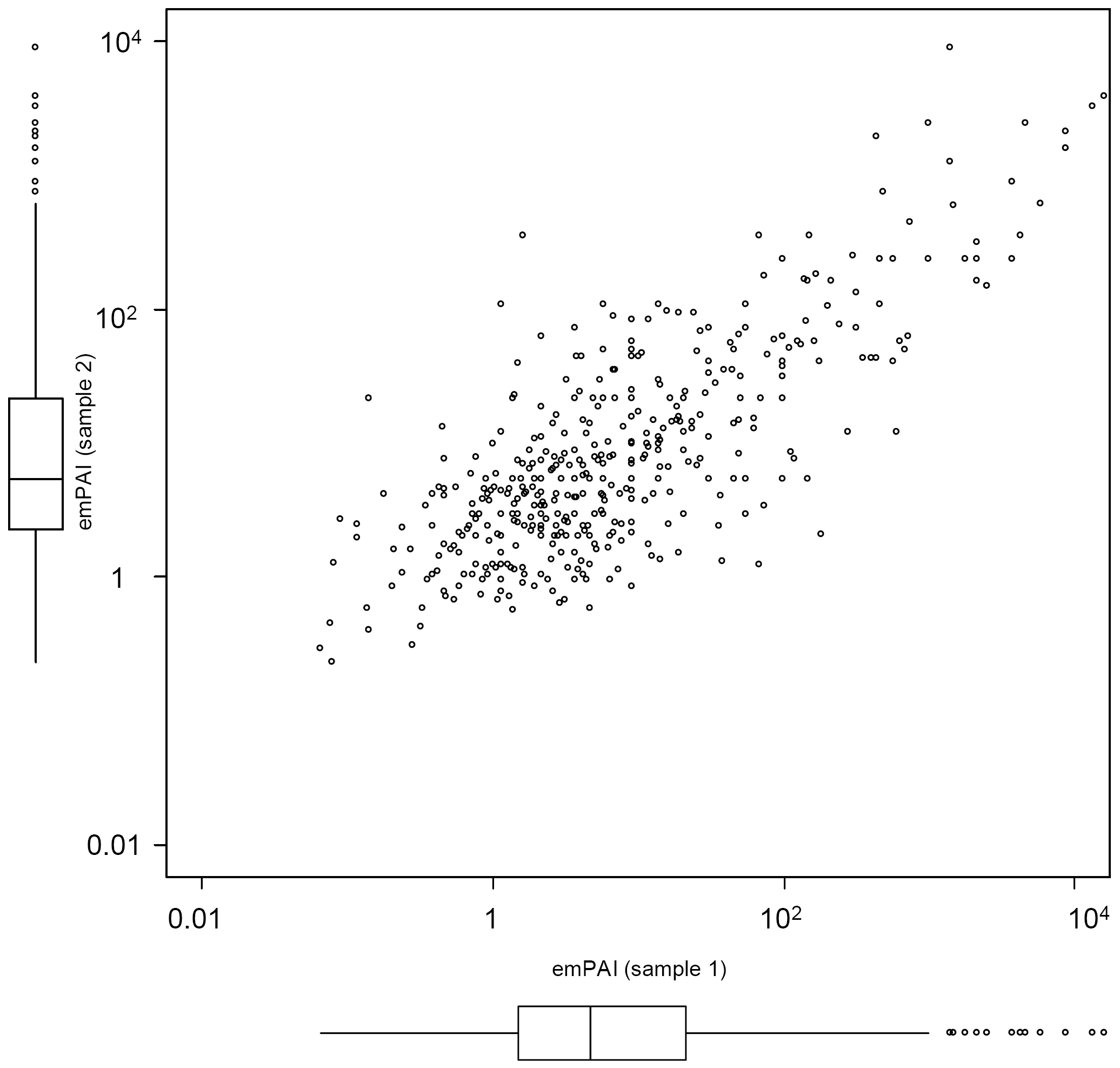
**

**Supplementary Figure S5:** *Reproducibility of emPAI values for replicate biological samples of E. coli cytosol.*

Comparison of emPAI values of 714 proteins with more than one identified peptide between two experiments performed with replicate preparations of the E. coli cytosol.

The Pearson correlation coefficient of the logarithmized variables is 0.78 with a p-value < 1E-88 and 0.54 (p-value <1E-32) of the original values.

**
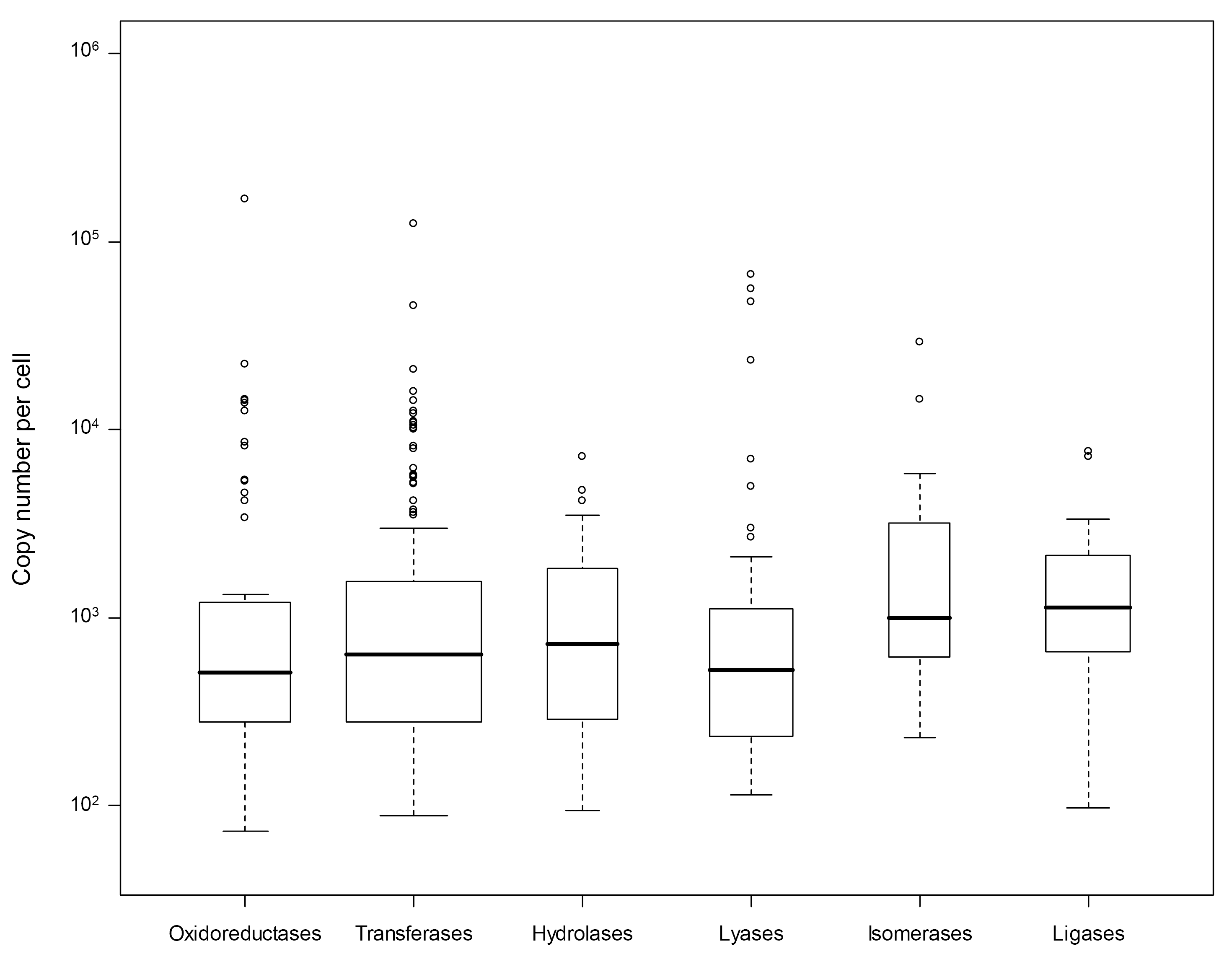
**

**Supplementary Figure S6:** *Abundance distribution of proteins classified according to the EC Enzyme classification scheme.*

The thick vertical bar shows the median abundance value of each group. The thickness of each boxplot represents the number of different proteins which belong to each class. Taking the median abundance for comparisons, ligases are the most abundant enzymes followed by isomerases.


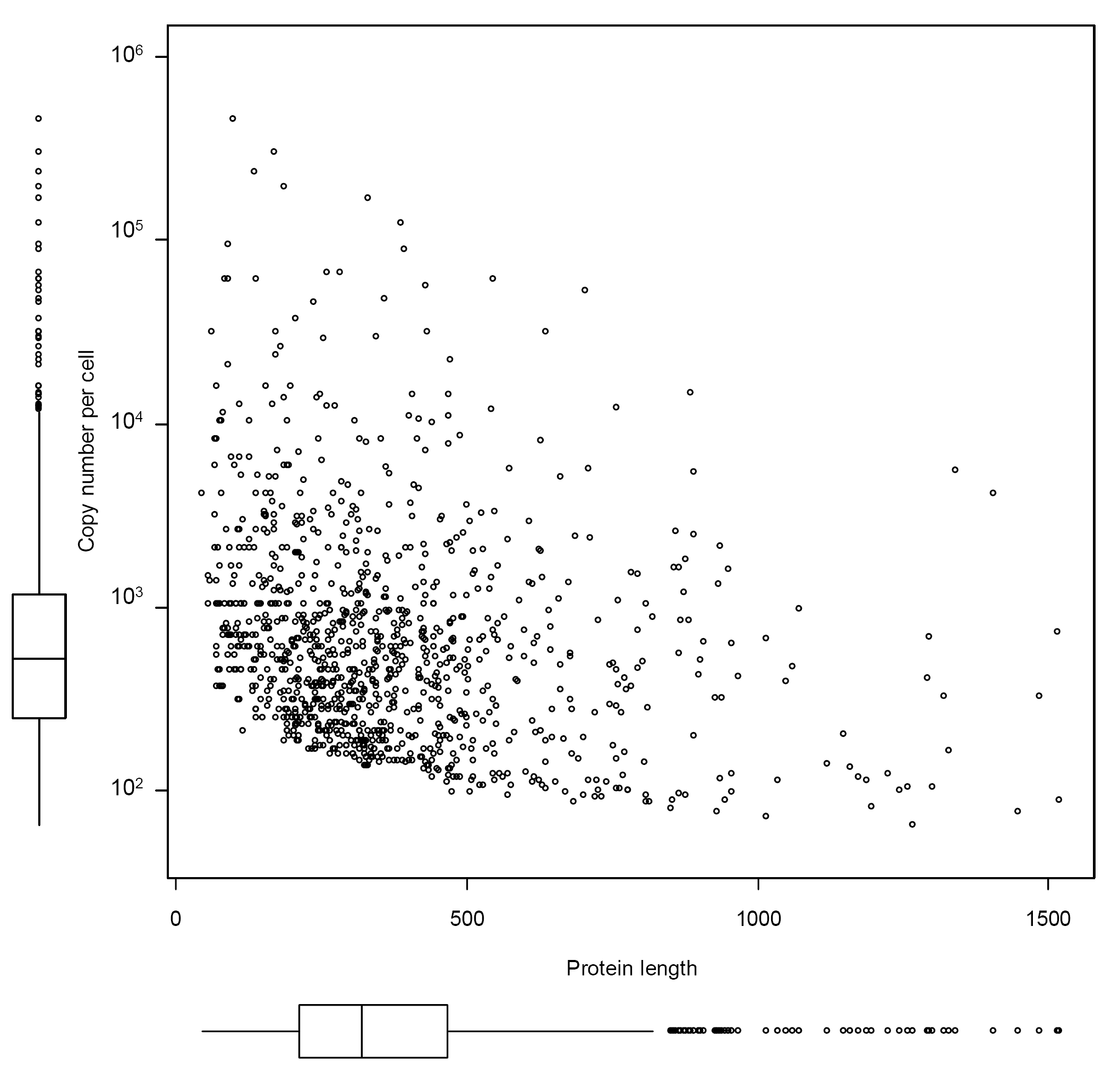


**Figure S7**: *Abundance vs. Protein length*

High abundant proteins tend to be short, in the right upper corner (abundant and long) no proteins can be found, whereas in the low abundant range multiple long proteins can be seen.

**4. Supplementary Materials and Methods**

### Protein and peptide fractionation

*SDS-PAGE/in-gel digestion*: The soluble portion of the spheroplast lysate solution (60 µg total proteins) was separated by SDS-PAGE (16%, 1.5 mm, 200V for 2 hrs). The gel was Coomassie brilliant blue stained, entire lanes were cut out and sliced into pieces, in-gel reduced, alkylated, and digested using trypsin (Promega, Madison, WI, USA) as described [8]. After extracting peptides from gel pieces using 3% TFA and 30% acetonitrile, the sample volume was partially reduced by vacuum evaporation and the residual solutions were applied to StageTip desalting [9] and /or peptide fractionation as described below.

*Subdivided scan range*: Divided MS scan range using QSTAR was performed as reported [10]. Briefly, the mass ranges for fragmentation were *m*/*z* = 350–550, *m*/*z* = 550–750 and *m*/*z* = 750–1400, and the target values for the pulsing enhancement were 400, 600 and 800, respectively.

*Ultrafiltration/in-solution digestion:* The soluble lysate (60 µg total proteins) was separated with Millipore ultrafiltration spin columns (cut-off: 100K, 50K, and 10K in series). The fractions were concentrated and re-suspended in 50 mM Tris-HCl buffer (pH 9.0) containing 8 M urea. These mixtures were subsequently reduced, alkylated, and digested with lysyl-endopeptidase (Wako, Osaka, Japan) and trypsin as described. Digested solutions were acidified with TFA, and were applied to StageTips.

*On-line SCX-RP fractionation*: The digested solutions were loaded onto a triphasic C18-SCX-C18 column for nanoLC-MS/MS and were fractionated using the second phase with the ammonium salt elution to the third phase followed by C18-based LC-MS/MS [11].

*StageTip fractionation:* All StageTips were prepared using a fully automated instrument (Nikkyo Technos, Tokyo, Japan) with Empore C18, PSDVB, SAX or SCX disks (3M, MN, USA). Peptide fractionation by SCX-StageTip was performed with 0-500 mM 4-step ammonium acetate salt elution, resulting fractions were desalted using C18-StageTips prior to LC-MS/MS analysis [6]. SAX-based fractionation was conducted using SAX-StageTips with pH-elution using 50mM ammonium carbonate, 50mM ammonium acetate and 0.1% TFA solutions. C18 StageTips with 0.1% dibutylammonium acetate (DBAA) as ion pair reagent were used for fractionation with different contents of acetonitrile. PSDVB-StageTips fractionation was performed on 0.1% ammonium hydroxide and a step gradient elution with acetonitrile.

### NanoLC-MS/MS Analysis

All samples were analyzed by nanoLC-MS/MS using a QSTAR Pulsar i (AB/MDS-Sciex, Toronto, Canada), or a Finnigan LTQ (Thermoelectron, San Jose, CA, USA) equipped with a Agilent1100 nanoflow pump (Germany), and an HTC-PAL autosampler (CTC Analytics AG, Zwingen, Switzerland) equipped with Valco C2 valves with 150 µm ports. ReproSil C18 materials (3 µm, Dr. Maisch, Ammerbuch, Germany) were packed into a self-pulled needle (100 µm ID, 6 µm opening, 150 mm length) with a nitrogen-pressurized column loader cell (Nikkyo) to prepare an analytical column needle with "stone-arch" frit [12]. A Teflon-coated column holder (Nikkyo) was mounted on an x-y-z nanospray interface (Proxeon, Odense, Denmark) and a Valco metal connector with a magnet was used to hold the column needle and to set the appropriate spray position. The injection volume was 2.5 µL and the flow rate was 500 nL/min. The mobile phases consisted of (A) 0.5% acetic acid and (B) 0.5% acetic acid and 80% acetonitrile. The three-step linear gradient of 5% B to 10% in 5 min, 10% to 30% in 60 min, 30% to 100% in 5 min and 100% in 10 min were employed '60 min gradient' throughout this study except when described differently. A spray voltage of 2400 V was applied via the metal connector as described [12]. For QSTAR experiments with the faster scan mode, MS scans were performed for 1 second to select three intense peaks and subsequently three MS/MS scans were performed for 0.55 seconds each. An Information Dependent Acquisition (IDA) function was active for three minutes to exclude the previously scanned parent ions. For LTQ experiments, two MS/MS scans per one MS scan were performed in the automated gain control (AGC) mode. The scan cycle was 0.17 s for one MS and 0.38 s for one MSMS on average in LTQ. The scan range was m/z 350-1400 for QSTAR and LTQ.

**5. References**

1. Taoka M, Yamauchi Y, Shinkawa T, Kaji H, Motohashi W, Nakayama H, Takahashi N, Isobe T: **Only a small subset of the horizontally transferred chromosomal genes in Escherichia coli are translated into proteins**. *Mol Cell Proteomics* 2004, **3**(8):780-787.

2. Kristensen DB, Brond JC, Nielsen PA, Andersen JR, Sorensen OT, Jorgensen V, Budin K, Matthiesen J, Veno P, Jespersen HM *et al*: **Experimental Peptide Identification Repository (EPIR): an integrated peptide-centric platform for validation and mining of tandem mass spectrometry data**. *Mol Cell Proteomics* 2004, **3**(10):1023-1038.

3. Kerner MJ, Naylor DJ, Ishihama Y, Maier T, Chang HC, Stines AP, Georgopoulos C, Frishman D, Hayer-Hartl M, Mann M *et al*: **Proteome-wide analysis of chaperonin-dependent protein folding in Escherichia coli**. *Cell* 2005, **122**(2):209-220.

4. Spahr CS, Davis MT, McGinley MD, Robinson JH, Bures EJ, Beierle J, Mort J, Courchesne PL, Chen K, Wahl RC *et al*: **Towards defining the urinary proteome using liquid chromatography-tandem mass spectrometry. I. Profiling an unfractionated tryptic digest**. *Proteomics* 2001, **1**(1):93-107.

5. Ishihama Y: **Proteomic LC-MS systems using nanoscale liquid chromatography with tandem mass spectrometry**. *J Chromatogr A* 2005, **1067**(1-2):73-83.

6. Ishihama Y, Rappsilber J, Mann M: **Modular stop and go extraction tips with stacked disks for parallel and multidimensional Peptide fractionation in proteomics**. *J Proteome Res* 2006, **5**(4):988-994.

7. Havlis J, Shevchenko A: **Absolute quantification of proteins in solutions and in polyacrylamide gels by mass spectrometry**. *Anal Chem* 2004, **76**(11):3029-3036.

8. Lasonder E, Ishihama Y, Andersen JS, Vermunt AM, Pain A, Sauerwein RW, Eling WM, Hall N, Waters AP, Stunnenberg HG *et al*: **Analysis of the Plasmodium falciparum proteome by high-accuracy mass spectrometry**. *Nature* 2002, **419**(6906):537-542.

9. Rappsilber J, Ishihama Y, Mann M: **Stop and go extraction tips for matrix-assisted laser desorption/ionization, nanoelectrospray, and LC/MS sample pretreatment in proteomics**. *Anal Chem* 2003, **75**(3):663-670.

10. Rappsilber J, Ryder U, Lamond AI, Mann M: **Large-scale proteomic analysis of the human spliceosome**. *Genome Research* 2002, **12**(8):1231-1245.

11. Link AJ, Eng J, Schieltz DM, Carmack E, Mize GJ, Morris DR, Garvik BM, Yates JR, 3rd: **Direct analysis of protein complexes using mass spectrometry**. *Nat Biotechnol* 1999, **17**(7):676-682.

12. Ishihama Y, Rappsilber J, Andersen JS, Mann M: **Microcolumns with self-assembled particle frits for proteomics**. *Journal of chromatography* 2002, **979**(1-2):233-239.
